# Inhibin Immunization to Enhance Reproductive Performance in Livestock: A Review

**DOI:** 10.3390/biology15070528

**Published:** 2026-03-26

**Authors:** Abd Ullah, Muhammad Zahoor Khan, Changfa Wang

**Affiliations:** College of Agriculture and Biology, Liaocheng University, Liaocheng 252000, China; 2024090034@stu.lcu.edu.cn

**Keywords:** inhibin, FSH, superovulation, embryo production, spermatogenesis, livestock reproductive biotechnology, immunization

## Abstract

Reproductive efficiency substantially determines livestock productivity, and suboptimal fertility continues to limit productivity across species. Inhibin immunization represents a novel strategy to enhance fertility by neutralizing inhibin’s negative feedback on follicle-stimulating hormone (FSH) secretion. This comprehensive review synthesizes evidence from ruminants (cattle, sheep, goats, camels, and buffalo) and non-ruminants (pigs, donkeys, rabbits, and avian species), evaluating inhibin immunization mechanisms and reproductive outcomes. Immunoneutralization of inhibin consistently elevates FSH, activin A, and estradiol, thereby promoting ovarian follicular recruitment and spermatogenesis. In females, this approach has been widely reported to increase ovulatory activity and improve embryo production and quality. In males, immunization enhances testicular function and semen quality while mitigating seasonal infertility. However, species-specific variations in response and dose-dependent luteal suppression (particularly in pigs) necessitate optimized protocols. While inhibin immunization shows consistent benefits when integrated with multiple ovulation and embryo transfer (MOET) and in vitro fertilization (IVF), standardized protocols and long-term fertility data remain insufficient for commercial implementation. This review identifies critical evidence gaps and safety considerations essential for clinical translation.

## Simple Summary

Farmers and breeders constantly seek ways to produce more offspring from high-value animals. One underexplored approach involves vaccinating livestock against inhibin, a hormone that naturally limits reproductive activity. By training the immune system to block inhibin, animals produce more eggs, conceive more readily, and males generate better-quality sperm. This review examines how this vaccination strategy works across a wide range of farm animals, from cattle and sheep to camels, pigs, and donkeys. While results are encouraging, responses differ considerably between species, and some animals require additional hormonal support after vaccination. Broader trials and standardized guidelines are still needed before widespread farm adoption becomes feasible.

## 1. Introduction

**Figure 1:**
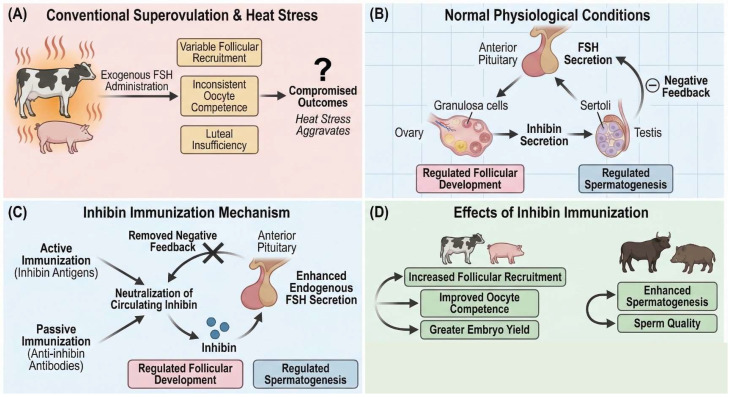
Schematic representation of the inhibin immunization mechanism and its effects on reproductive efficiency in livestock. (**A**) Conventional superovulation protocols utilizing exogenous FSH administration result in variable follicular recruitment, inconsistent oocyte competence, and luteal insufficiency, with outcomes further compromised by heat stress in both ruminants and non-ruminants. (**B**) Under normal physiological conditions, inhibin secreted by ovarian granulosa cells in females and testicular Sertoli cells in males exerts negative feedback on anterior pituitary FSH secretion, thereby regulating follicular development and spermatogenesis, respectively. (**C**) Inhibin immunization, achieved through either active immunization with inhibin antigens or passive administration of anti-inhibin antibodies, neutralizes circulating inhibin and removes negative feedback inhibition on pituitary FSH release. (**D**) Enhanced endogenous FSH secretion following inhibin immunization promotes increased follicular recruitment, improved oocyte competence, and greater embryo yield in females while supporting enhanced spermatogenesis and sperm quality in males. Comparative evaluation between ruminant and non-ruminant species remains essential for identifying translational pathways and addressing remaining knowledge gaps. FSH, follicle-stimulating hormone; MOET, multiple ovulation and embryo transfer; IVF, in vitro fertilization.

Suboptimal reproductive efficiency limits livestock productivity and genetic gain across species [1,2]. Conventional superovulation (SOV) protocols relying on repeated exogenous follicle-stimulating hormone (FSH) administration produce variable follicular recruitment, inconsistent oocyte competence, and luteal insufficiency, particularly in heat-stressed ruminants and non-ruminants [3,4]. These limitations constrain embryo yield and transfer success, reducing the efficiency of assisted reproductive technologies (ARTs) such as MOET and IVF [5,6,7]. Inhibin, a dimeric glycoprotein secreted by granulosa cells in females and Sertoli cells in males, exerts negative feedback on pituitary FSH secretion, thereby regulating follicular development and spermatogenesis [8,9,10,11]. By neutralizing inhibin’s suppressive effects through active or passive immunization, endogenous FSH secretion increases, promoting enhanced gonadal function independent of exogenous gonadotropins [12,13,14,15,16]. While inhibin immunization demonstrates fertility-enhancing effects in isolated species, a comprehensive comparative evaluation between ruminants and non-ruminants remains lacking. This review synthesizes evidence on inhibin biology, immunization mechanisms, and reproductive responses across species to identify translational pathways and remaining knowledge gaps. The underlying mechanism of inhibin immunization and its consequent impact on reproductive efficiency in livestock is summarized in Figure 1.

## 2. Inhibin Biology and Reproductive Regulation

**Figure 2:**
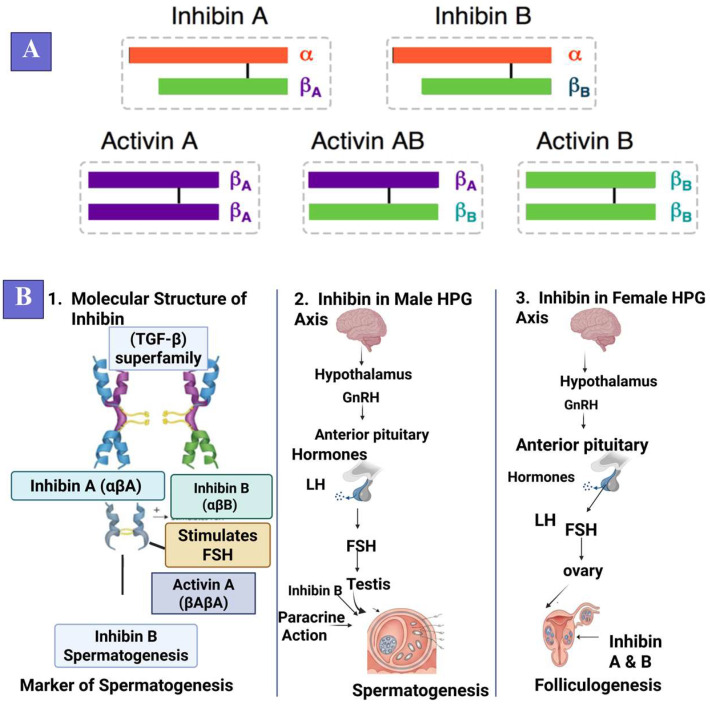
Molecular structure and role of inhibin in the male and female HPG axis. This figure illustrates the molecular structure of inhibin A and inhibin B, both of which belong to the TGF-β superfamily, consisting of an α-subunit and either a βA or βB subunit. The figure also includes activin A and activin AB, which influence FSH secretion. Panel (**A**) shows the molecular structure of these molecules, with the colored bars representing the relative size of each subunit. The red bars represent the α-subunit, and the green or purple bars represent the βA or βB subunits, respectively. The length of the colored bars reflects the size of each subunit in the structure. Panel (**B**) highlights the role of inhibin in the male and female HPG axis, where it regulates FSH secretion and thus plays a critical role in spermatogenesis in males and folliculogenesis in females. In males, inhibin B suppresses FSH to support testicular function, while in females, inhibin A and B regulate FSH during follicular development, promoting granulosa cell proliferation and inhibiting follicular atresia. This feedback mechanism is essential for reproductive regulation in both sexes.

Inhibin, belonging to the transforming growth factor-beta (TGF-β) superfamily, consists of an α-subunit paired with either βA or βB subunits, forming inhibin A and inhibin B [17,18,19,20,21]. The α-subunit contains N-linked glycosylation sites essential for bioactivity and FSH receptor binding [22]. Inhibin B, primarily secreted by Sertoli cells, serves as a reliable marker of spermatogenic activity, with circulating concentrations inversely related to FSH levels [21,23]. In females, inhibin concentrations increase during follicular wave emergence, directly correlating with follicular development and estradiol production [19,20,21]. Within the hypothalamic–pituitary–gonadal (HPG) axis, gonadotropin-releasing hormone (GnRH) from the hypothalamus stimulates pituitary release of luteinizing hormone (LH) and FSH [8,9]. FSH stimulates Sertoli cells and granulosa cells, which subsequently secrete inhibin, providing negative feedback suppression of FSH release, thereby controlling gonadal function [8]. Excessive inhibin concentrations suppress follicular wave progression and impair oocyte competence, reducing fertility and embryo output [12,13]. Activin A, a related TGF-β family member, opposes inhibin effects by stimulating rather than suppressing FSH secretion, with elevated activin levels accompanying inhibin neutralization [13,15,24,25,26]. In addition, as a key modulator of circulating follicle-stimulating hormone concentrations, inhibin governs the ovulatory cascade and follicular recruitment dynamics, processes that fundamentally determine prolificacy in ruminants [27,28]. The molecular structure of Inhibin A and B and their regulatory role in the male and female HPG axes are depicted in Figure 2.

## 3. Mechanism of Inhibin Immunization

Inhibin immunization elicits polyclonal anti-inhibin IgG antibodies that neutralize endogenous inhibin through receptor-blocking, immune complex formation and enhanced antibody-dependent cellular cytotoxicity (ADCC). Typical immunization protocols employ synthetic inhibin peptides (1–33 N-terminal α-subunit sequences) conjugated to carrier proteins (rabbit serum albumin), emulsified with Freund’s complete adjuvant, and administered intramuscularly with boosters at specified intervals [29,30]. Booster immunizations generate secondary responses with 10–100-fold higher titers within 7–14 days, with sustained titers persisting 4–8 weeks [13,15,24,31]. Immunoneutralization of inhibin relieves FSH suppression, increasing pituitary FSH secretion through relief of Smad-2/3 phosphorylation at FSH-β promoter elements [13,24,31]. Elevated FSH activates cAMP/PKA and PI3K/Akt signaling cascades in Sertoli cells and granulosa cells, promoting proliferation and enhanced expression of transcription factors (c-Myc, cyclin D1) [32,33,34,35]. FSH additionally promotes secretion of growth factors (GDNF, FGF2) essential for spermatogonia self-renewal and proliferation [8,36,37]. Importantly, inhibin immunization simultaneously elevates circulating activin A, which synergistically stimulates Sertoli cell proliferation through Smad-mediated signaling [38,39]. In contrast to the robust and consistent rise in FSH, LH responses to inhibin immunization are typically small and variable, with some studies reporting no significant change and others showing modest increases depending on protocol and physiological context [40,41]. This highlights the need for longitudinal endocrine monitoring to clarify compensatory HPG-axis adaptations.

**Figure 3:**
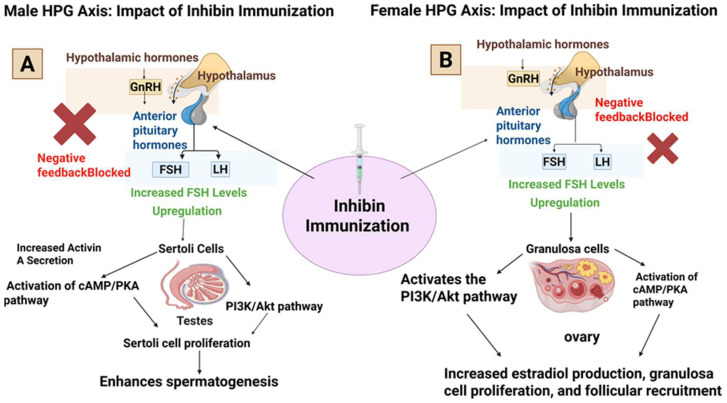
(**A**) Male HPG axis: impact of inhibin immunization. This diagram illustrates the effects of inhibin immunization on the male hypothalamic–pituitary–gonadal (HPG) axis. Immunization blocks the negative feedback loop, leading to increased secretion of follicle-stimulating hormone (FSH) and luteinizing hormone (LH). This results in enhanced Sertoli cell function, increased spermatogenesis, and improved sperm production. (**B**) Female HPG axis: impact of inhibin immunization. This diagram depicts the effects of inhibin immunization on the female HPG axis. Immunization against inhibin blocks its negative feedback, leading to increased FSH secretion. This enhances follicular development, increases oocyte yield, and improves ovulation rates and estrogen production, thereby enhancing reproductive performance.

Inhibin immunization has distinct effects on both male and female reproductive performance by disrupting the negative feedback mechanism in the hypothalamic–pituitary–gonadal axis, resulting in enhanced spermatogenesis in males and improved follicular development and ovulation in females in Figure 3. Although inhibin immunization clearly elevates FSH and modulates downstream signaling pathways, the long-term regulation of the hypothalamic–pituitary–gonadal (HPG) axis following inhibin immunoneutralization remains insufficiently understood. Therefore, further studies are required to clarify the endocrine adaptations and long-term reproductive consequences of this strategy.

## 4. Reproductive Responses to Inhibin in Livestock

Female reproductive performance is regulated by dynamic interactions within the hypothalamic–pituitary–gonadal axis, and disruptions in this system can impair follicular development, ovulation, oocyte quality, and overall fertility [42,43]. FSH plays a central role in female reproduction by stimulating granulosa cell proliferation, follicular growth, aromatase activity, and estradiol synthesis, thereby supporting dominant follicle selection and oocyte maturation [44,45]. Inhibin, a dimeric glycoprotein secreted primarily by ovarian granulosa cells, exerts negative feedback on pituitary FSH secretion and helps regulate follicular wave dynamics [46]. Immunoneutralization of inhibin relieves this negative feedback, thereby enhancing endogenous FSH secretion and promoting follicular recruitment, estradiol production, ovulation, and embryo yield.

Male reproductive performance declines with age due to progressive alterations within the hypothalamic–pituitary–gonadal axis, leading to reduced semen quality and diminished endocrine function [47,48,49]. FSH is central to male fertility, acting directly on Sertoli cells to stimulate germ cell proliferation and indirectly on Leydig cells to enhance androgen synthesis, thereby supporting spermatogenesis [50]. Inhibin, a dimeric glycoprotein secreted primarily by Sertoli cells, exerts negative feedback on pituitary FSH secretion and modulates GnRH/LH release at the hypothalamic level [30]. Among its isoforms, inhibin B serves as the predominant endocrine regulator in males, with circulating concentrations closely reflecting Sertoli cell number, testicular development, and spermatogenic activity. Its characteristic developmental pattern—postnatal elevation, prepubertal decline, and pubertal rise—makes inhibin B a reliable biomarker for assessing testicular function and for monitoring reproductive responses to inhibin immunization [8,51,52,53]. Immunoneutralization of inhibin enhances endogenous FSH secretion, promoting Sertoli cell function and spermatogonial development through key intracellular pathways. Increased FSH activates the cAMP/PKA signaling cascade, which regulates genes essential for Sertoli cell activity and spermatogenesis [34,54], while elevated activin further stimulates Sertoli cell proliferation and survival through Smad-mediated signaling [38,39]. The following sections present the effects of inhibin immunization in ruminant and non-ruminant species.

### 4.1. Reproductive Responses in Female Ruminants

In female ruminants, inhibin immunization has emerged as an effective strategy to enhance ovarian activity, superovulation, and embryo production by counteracting the suppressive effects of endogenous inhibin [13,18]. This immunization results in elevated follicle-stimulating hormone (FSH) levels, promoting follicular development and improving reproductive outcomes [15,55]. Han et al. [27] discuss how TGF-β signaling, including genes such as BMP15, GDF9, and BMPR1B, regulates litter size in sheep and goats by enhancing ovarian sensitivity to gonadotropins, thereby increasing ovulation rates. Hen et al. [28] review genetic markers influencing litter size in goats, highlighting key genes such as BMPs, GDF9, and SMAD family members. These genes regulate critical processes such as folliculogenesis, ovulation, and hormone regulation, offering valuable insights for breeding programs aimed at improving reproductive efficiency. Studies have demonstrated significant enhancements in reproductive parameters, including ovulation rates, follicular recruitment, and embryo quality, as summarized in Table 1, with variations across species, immunogen types, and reproductive contexts.

#### 4.1.1. Cattle

In cattle, active and passive inhibin immunization strategies can be integrated with conventional SOV protocols [14,15,55,56,57]. In Japanese beef heifers, re-immunization against inhibin sustained circulating FSH without affecting LH, resulting in increased estradiol (3–4-fold) and progesterone secretion, with ovulation rates increasing from 1 to approximately 5 [14]. When combined with exogenous porcine FSH, immunized Japanese Black cows exhibited significantly higher follicle numbers, corpora lutea numbers, and embryo yields (12.1 ± 1.2 total embryos vs. 8.2 ± 1.0 control; 5.7 ± 1.1 transferable embryos vs. 3.1 ± 0.7 control) [55]. Meta-analytic evidence demonstrates that recombinant inhibin DNA vaccines significantly increased ovulation numbers, embryo yields, and conception rates in Holstein, Friesian, and Hereford cattle, especially when combined with standard SOV protocols [13]. In Holstein cows, dose-dependent increases in FSH, estradiol, and activin A accompanied improved follicular development, though some progesterone suppression during the luteal phase was noted, indicating the need for protocol optimization [15]. In water buffalo, combining inhibin immunization with Ovsynch improved follicular development, estrus expression, and ovulation rate (91% vs. 54%) [58].

#### 4.1.2. Sheep and Goats

In sheep, inhibin immunization has been explored to enhance reproductive performance, increasing ovulation rates and embryo production. In Tan sheep, DNA vaccines encoding inhibin (1–32) alone or fused with RF-amide-related peptide-3 (RFRP-3) induced strong immune responses and significantly elevated circulating FSH, LH, and estradiol [59]. In Kazakh sheep during anestrus, both recombinant inhibin-α protein and camel-derived anti-inhibin antibodies significantly increased FSH and estradiol without altering LH or progesterone levels [60]. Most substantial gains occurred when FSH was co-administered with immunization: ovulation rates increased from 5.0 in conventionally super-ovulated ewes to 12.1 in immunized ewes, with embryo quality improving markedly (6.7 vs. 1.5 transferable embryos) [61]. In Awassi ewes, both active and passive inhibin immunization, combined with progesterone synchronization or eCG, increased estradiol, FSH, and LH levels, embryo numbers, and lambs born per ewe [62].

In goats, inhibin immunization has consistently shown positive effects on reproductive performance across various breeds and management systems [63,64,65,66,67]. In Boer goats, immunization led to robust follicular recruitment and consistent ovulation numbers (≈9 ovulations per doe; embryo yield ≈5.7 per doe) even during an anestrous period [63]. Efficacy was modulated by the endocrine context: maintaining goats in a subluteal progesterone environment significantly enhanced the superovulatory response to passive immunization, doubling the ovulation rate (13.9 vs. 5.7) [68]. In Beetal goats, 0.5 mg inhibin immunization increased follicle size, ovulatory follicle diameter, luteal development, and prolificacy while maintaining pregnancy rates [69].

#### 4.1.3. Camels

Camels exhibit unique reproductive challenges, as their performance is highly sensitive to environmental and physiological stressors, particularly heat stress and nutritional limitations, which can disrupt endocrine function and impair fertility [70]. In this context, inhibin immunization has emerged as a promising strategy to enhance ovarian activity and improve fertility in female dromedaries. Reproductive performance in female dromedaries is highly sensitive to environmental and physiological stressors, particularly heat stress and nutritional limitations, which can disrupt endocrine function and impair fertility. Subfertile camels exhibit characteristic hormonal and inflammatory alterations, with inhibin, FSH, and nitric oxide (NO) identified as reliable biomarkers of reproductive dysfunction [71]. Elevated inhibin and IL-1β levels have been specifically associated with cervicitis and vaginitis, underscoring the contribution of inflammatory pathways to reduced fertility. Against this backdrop, immunological manipulation of inhibin has emerged as a promising strategy for improving ovarian activity in camels. Active immunization against inhibin produced a pronounced and sustained ovarian hyperstimulation lasting up to five months post-vaccination, characterized by elevated FSH concentrations, increased numbers of growing and preovulatory follicles, and a 100% triple-ovulation rate—an outcome comparable to eCG-based superovulation protocols but achieved without exogenous gonadotropins [72].

Additional endocrine-based reproductive control methods, such as the anti-GnRF vaccine Improvac, effectively suppress estrus in Iberian pigs, reducing progesterone concentrations and reproductive organ size and offering a non-surgical tool for managing fertility and preventing mating with wild boars [73]. Overall follicular dynamics in pigs depend on the coordinated interplay among FSH, LH, inhibin, estradiol, and metabolic hormones; these interactions govern the weaning-to-estrus interval, a critical determinant of reproductive efficiency in sows [74].

Further evidence supports the utility of inhibin immunization in overcoming seasonal constraints on camel reproduction. In dromedaries transitioning through seasonal anestrus, active immunization against inhibin A increased circulating FSH and estradiol and promoted continuous follicular recruitment and growth, thereby improving ovarian responsiveness during periods of naturally low activity [75]. Alongside immunization approaches, other reproductive enhancement strategies—including melatonin supplementation, manipulation of photoperiod, and ovarian superstimulation with FSH or eCG—have been employed to extend the breeding season and improve embryo recovery. However, superstimulation protocols in camels are often limited by variable responses, follicular luteinization, and inconsistent ovulatory outcomes, highlighting the need for more reliable alternatives [76]. Although inhibin immunization shows considerable promise as a long-lasting and physiologically targeted strategy, more comprehensive studies are still required to optimize dosing, evaluate long-term fertility outcomes, and assess its application across different camel breeds and production settings.

**Table 1:** Effects of inhibin immunization on reproductive parameters in female ruminants.

Species	Immunogen Type	Antibody Titer/FSH Change	Reproductive Outcome	Key Findings	Reference
Cattle (Japanese beef heifers)	Recombinant ovine inhibin α-subunit vaccine in oil emulsion; three boosters (one year after primary immunization)	Rapid high anti-inhibin antibody titers within 9 days; sustained titers; FSH ↑, LH ↔	↑ Estradiol (3–4×), ↑ progesterone, ↑ follicles (all sizes), ovulation rate ≈5 vs. 1 control, earlier estrus post-PGF_2_α.	Re-immunization induces repeatable superovulation without exogenous FSH; enhances oocyte supply for IVF and embryo transfer	[14]
Holstein cows	Recombinant porcine inhibin α-subunit protein (0.5 mg and 1 mg doses)	Dose-dependent increase in anti-inhibin antibody titers, elevated FSH, E2, and activin A	Increased conception rates (high-dose: 45.5%, low-dose: 40%)	Immunization improved conception rates, stimulated follicular development, and increased FSH, E2, and activin A levels; however, it compromised luteal function, as indicated by reduced P4 levels.	[15]
Cattle (Japanese black cows)	Active immunization with porcine inhibin α-subunit fragment (1–26) conjugated to rabbit serum albumin, with boosters at 35 and 70 days	High anti-inhibin antibody titers; enhanced FSH responsiveness	↑ Follicles and corpora lutea; total embryos 12.1 ± 1.2 vs. 8.2 ± 1.0; transferable embryos 5.7 ± 1.1 vs. 3.1 ± 0.7 (control)	Immunization amplified ovarian response to exogenous FSH, improving superovulation and embryo yield.	[55]
Sheep (Tan ewes)	DNA vaccines encoding inhibin (1–32) alone (p-SINH) or fused with RFRP-3 (p-TPA-SINH/TPA-SRFRP, p-SINH/SRFRP); three i.m. injections 20 d apart	All vaccine groups developed strong anti-INH (and, where applicable, anti-RFRP-3) IgG; FSH and LH significantly ↑ in fusion-vaccine groups vs. control	Twinning rate numerically ↑ (37.5%, 37.5%, 12.5% vs. 0%) but not statistically significant	Fusion INH(1–32)/RFRP-3 DNA vaccines successfully neutralized endogenous INH and RFRP-3 and elevated FSH, LH, and E2.	[59]
Kazakh sheep	Camel-derived anti-INHα polyclonal antibody; recombinant INHα protein	Group A (anti-INHα antibody): FSH and estradiol significantly increased; endogenous inhibin decreased; LH and progesterone unchanged. Group B (recombinant INHα): no significant hormonal change vs. control.	Direct fertility indices not measured; study conducted during anestrus	Neutralization of inhibin-α effectively elevates FSH and E2 without disrupting LH, P4, or blood biochemical parameters, demonstrating its potential as an immunological approach to improve fecundity in Kazakh sheep.	[60]
Sheep (Awassi ewes)	Active: synthetic inhibin-α peptide conjugated to ovalbumin; passive: steroid-free bovine follicular fluid antiserum (SFBFF)	Inhibin neutralized; FSH not directly measured, but increased endogenous FSH secretion inferred via removal of inhibin negative feedback	↑ Embryo number (≈2.2 vs. 1.0–0.6 in controls), ↑ lambs per ewe, ↑ progesterone and ↓ estradiol during gestation in SI and AI groups vs. controls	Both active (synthetic inhibin) and passive (SFBFF antiserum) immunization during the non-breeding season enhanced ovulation rate and litter size in Awassi ewes, indicating that inhibin immunoneutralization can augment fecundity without exogenous gonadotropins.	[62]
Goat (Boer does)	Recombinant ovine inhibin α-subunit (primary + booster, oil-based adjuvant)	Strong antibody response; peak titer 2 weeks after booster; increased follicular recruitment independent of exogenous gonadotropins	≈22 large follicles, ≈9 ovulations per doe; embryo yield ≈5.7 per doe; all does show estrus post-immunization even with saline treatment.	Inhibin immunization alone induced robust superovulation; exogenous eCG or pFSH no longer increased the response; suggests dominant intra-ovarian paracrine role of inhibin; viable alternative to conventional gonadotropin superovulation	[63]
Goat (Shiba, Japan)	Passive i.v. inhibin antiserum (10 mL), evaluated under subluteal vs. normal luteal P_4_	Inhibin binding ↑ in both groups; LH and E2 ↑ under subluteal P_4_; FSH similar	Sub-luteal P_4_ markedly ↑ follicle number, follicle size, and ovulation rate (13.9 vs. 5.7)	Sub-luteal progesterone greatly enhances the superovulatory response to inhibin neutralization.	[68]
Beetal goats	Active immunization with recombinant inhibin α-subunit (0, 0.5, or 1 mg; two doses before estrus induction)	Effective immune response; 0.5 mg dose increased follicle size, pre-ovulatory follicle diameter, CL size, and post-breeding P4	Pregnancy rate unchanged; prolificacy and twinning rate higher in 0.5 mg group; fetal/embryonic loss unchanged; kid birth weight lower in immunized groups	0.5 mg dose optimizes ovarian dynamics and prolificacy during progestin-induced estrus in non-breeding season; no benefit of 1 mg dose; supports inhibin immunization as an adjunct to reproductive management	[69]
Dromedary camel	Active immunization against recombinant bovine inhibin A; 1 mL initial dose + two boosters at 14-day intervals	↑ FSH (≈3.3× control), ↑ estradiol (≈2.6× control), ↓ circulating inhibin A	↑ Total follicle number (≈3.5× control), ↑ dominant follicle size, sustained follicular growth throughout transition period	Inhibin immunization enhances ovarian activity and helps override seasonal anestrus.	[75]
Camel (dromedary females)	Recombinant bovine inhibin A vaccine (100 μg s.c. + two 50 μg boosters; evaluated 5 months later under timed-ovulation protocol)	Higher FSH than eCG-treated and control camels; circulating inhibin A and E2 ↑ in immunized group	↑ Total follicles (9.0 ± 1.0 vs. 3.0 ± 0.7 control); triple-ovulation rate 100% (vs. 0% control); dominant follicle size ↔	Immunization produced prolonged ovarian hyperactivity and enabled reliable timed ovulation; effective alternative to eCG-based superovulation in camels	[72]

**Explanation for Arrows: ↑**: Increase in parameter. **↓**: Decrease in parameter. **↔**: No significant change.

### 4.2. Reproductive Responses in Male Ruminants

Male reproductive performance declines with age due to progressive alterations within the hypothalamic–pituitary–gonadal axis, leading to reduced semen quality and diminished endocrine function [47,48,49]. FSH plays a critical role in male fertility by acting on Sertoli cells to stimulate germ cell proliferation and indirectly on Leydig cells to enhance androgen synthesis, supporting spermatogenesis [50]. Inhibin, a dimeric glycoprotein secreted primarily by Sertoli cells, exerts negative feedback on pituitary FSH secretion and modulates GnRH/LH release at the hypothalamic level [30]. Among its isoforms, inhibin B serves as the predominant endocrine regulator in males, with circulating concentrations closely reflecting Sertoli cell number, testicular development, and spermatogenic activity. Its characteristic developmental pattern—postnatal elevation, prepubertal decline, and pubertal rise—makes inhibin B a reliable biomarker for assessing testicular function and for monitoring reproductive responses to inhibin immunization [8,51,52,53]. Immunoneutralization of inhibin enhances endogenous FSH secretion, promoting Sertoli cell function and spermatogonial development through key intracellular pathways. Increased FSH activates the cAMP/PKA signaling cascade, which regulates genes essential for Sertoli cell activity and spermatogenesis [34,54], while elevated activin further stimulates Sertoli cell proliferation and survival through Smad-mediated signaling [38,39]. The following sections present the effects of inhibin immunization in male ruminant and non-ruminant species.

In male ruminants, inhibin immunization has shown consistent benefits in improving semen quality and stimulating testicular function [77,78]. The following subsections explore the effects of inhibin immunization in bucks, boars, and donkeys, with specific attention to the hormonal and testicular responses as well as the practical outcomes for breeding. The observed effects on male reproductive physiology highlight the potential of inhibin immunization as a tool to enhance fertility in ruminant livestock. These findings are summarized in Table 2, which outlines the influence of inhibin immunization on male ruminant hormones, testicular function, and semen quality.

#### 4.2.1. Bulls

Active immunization against inhibin-alpha in beef bulls increased serum FSH levels and testicular sperm density, suggesting that inhibin plays a key role in regulating spermatogenesis. However, immunization did not significantly affect testes weight or total daily sperm production, highlighting the potential of inhibin immunization to enhance fertility by modulating FSH secretion [79]. The study by Bame et al. [80] demonstrated that long-term immunization against inhibin in bulls led to increased sperm output, primarily through enhanced serum FSH concentrations. However, the response varied among individuals, with some bulls showing no significant improvement in sperm production. Another study found that inhibin immunoneutralization in bulls increased serum FSH and testosterone levels, decreased LH concentrations, and enhanced sperm production per gram of testicular parenchyma. However, testicular growth, scrotal circumference, and body weight were unaffected [81]. Inhibin immunoneutralization shows potential for enhancing sperm production by modulating FSH and testosterone levels in bulls; however, recent studies are limited in this area, necessitating further research to understand its effects on testicular growth, overall fertility, and its long-term impact on reproductive performance.

#### 4.2.2. Rams and Bucks

In male goats, inhibin immunization has demonstrated consistent endocrine and testicular effects across different breeds and management conditions. Passive immunization in sexually mature Shiba bucks increased plasma FSH and estradiol concentrations and significantly reduced resistance and pulsatility indices in the supratesticular and marginal testicular arteries, indicating improved testicular blood flow while leaving LH and testosterone unchanged [78]. In Chongming White goat lambs, repeated immunization with recombinant inhibin produced a transient rise in FSH and testosterone after the second booster, without affecting LH, growth traits, or testicular size; although testicular histology remained normal, gene-expression profiling revealed marked upregulation of Dmrtc2 and SOX30 and reduced AR expression, suggesting molecular-level modulation of Sertoli cell function and spermatogenic pathways.

Active inhibin-α immunization in Beetal bucks enhanced semen quality across both low and peak breeding seasons, improving sperm concentration, motility, viability, membrane and acrosome integrity, as well as post-thaw kinematic performance, indicating durable benefits extending into subsequent reproductive periods [77]. Similar findings in Shiba bucks showed that immunization induced a strong anti-inhibin antibody response and a temporary rise in FSH (5–9 weeks), accompanied by increased scrotal circumference and higher sperm concentrations, while semen volume and CASA-derived motility parameters remained comparable to controls [82]. In contrast, GnRH-based vaccines such as the GnRH6–kisspeptin–CRM197 conjugate elicit the opposite effect, dramatically reducing testosterone, shrinking testicular size, and impairing spermatogenesis, illustrating how immunization strategies can either enhance or suppress male fertility depending on the targeted hormone pathway [83]. Overall, inhibin immunization in bucks consistently improves testicular function and semen attributes; however, most studies have focused on conventional semen parameters. Further research is required to optimize immunization protocols and to evaluate long-term fertility outcomes, offspring performance, and advanced sperm quality indicators, such as DNA integrity and oxidative stress markers.

#### 4.2.3. Male Camels

In male dromedary camels, reproductive immunization strategies have produced varying physiological outcomes depending on the hormonal pathway targeted. Immunization against GnRH effectively reduces testosterone concentrations, suppresses libido, and impairs semen quality—particularly by decreasing acrosin amidase activity and increasing abnormal sperm morphology—while testicular volume remains largely unaffected [84]. Hormonal dysregulation is also evident in infertile camels: individuals with impotentia generandi (IG) show elevated serum FSH, LH, and testosterone associated with oligospermia or azoospermia, and smaller testes are accompanied by lower sperm counts and higher hormone levels, underscoring the diagnostic value of endocrine measurements in evaluating spermatogenic failure [85].

In contrast to GnRH suppression, inhibin immunization has shown promising fertility-enhancing effects. A recent study reported that immunized male camels developed strong anti-inhibin antibody titers, exhibited significantly increased FSH, estradiol, and nitric oxide levels, and demonstrated improved testicular blood flow, enlarged testicular size, and reduced tissue echogenicity—indicators consistent with active spermatogenesis and enhanced testicular function [30]. Sperm concentration and viability increased significantly after immunization, while LH, testosterone, and motility were unchanged, suggesting that inhibin neutralization can enhance fertility without disrupting androgen balance. However, because published data on inhibin immunization in male camels remain extremely limited, further controlled studies are needed to verify these findings, refine immunization protocols, and evaluate long-term reproductive outcomes.

**Table 2:** Effects of inhibin immunization on male reproductive parameters in ruminant.

Species	Immunogen Type	Hormonal Change	Testicular Response	Semen Quality	Reference
Goat (male, Shiba breed)	Passive i.v. immunization with 10 mL inhibin antiserum (raised in castrated goats against N-terminal α-chain of porcine inhibin)	Significant ↑ FSH from 60–144 h post-treatment; ↑ estradiol (E2) at 6, 12, and 36 h; no significant change in LH and testosterone	↓ Resistive index (RI) and pulsatility index (PI) in supratesticular and marginal testicular arteries at 24–120 h, indicating increased testicular blood flow/perfusion	Not evaluated/not reported in this study	[78]
Beetal bucks	Active immunization against inhibin α-subunit (0.5 mg primary + booster at day 28)	↑ Semen volume, ↑ sperm concentration, ↑ motility, ↑ viability, ↑ plasma membrane & acrosome integrity	↑ total & progressive motility, ↑ sperm kinetics, ↑ morphology; strong carry-over effect from LBS → PBS	Immunization improved fresh and post-thaw semen quality across seasons; reduced seasonal infertility effects	[77]
Ram lambs	α-inhibin peptide-ovalbumin conjugate (PTC), α-inhibin subunit (SUB)	Increased α-inhibin antibody titer (*p* < 0.0001) by day 14; plasma FSH increased (*p* = 0.02) in SUB-immunized group; LH and testosterone levels remained similar across all groups	Daily sperm production (DSP/g) increased by 26% (*p* < 0.01); total DSP per ram lamb did not differ; testes	No increase in total sperm production per ram	[86]
Bull calves	Immunization against inhibin α-subunit peptide (a-(1–25)-ha-G)	Plasma FSH concentrations increased significantly after inhibin immunization, especially at 60 and 120 days (*p* < 0.05).	inhibin production localized to Sertoli cells in seminiferous tubules. Testicular inhibin production detectable by Western blot at 7, 21, 60, and 120 days.	No significant change in scrotal circumference (SC) and sperm quality at pubertal onset	[87]
Bulls	Methimazole (antithyroid drug)	Decreased thyroid hormones (T3 and T4)	Increased Sertoli cell number (~2×), larger testes	Similar sperm motility, morphology, cryopreservation survival	[88]
Bulls	Transient hypothyroidism (Methimazole)	Decreased T3 and T4 levels	30–180% more sperm per ejaculate, 2.3-fold increase in Sertoli cells, higher testicular and epididymal weight	No effect on sperm motility, morphology, cryopreservation survival, or IVF success	[89]
Camel	Synthetic peptide (α-subunit 1–33 of porcine inhibin) conjugated to rabbit serum albumin; Freund’s complete adjuvant; 4 booster doses (weeks 0, 4, 8, 12)	↑ Anti-inhibin antibody titer; ↑ FSH; ↑ estradiol (E2); ↑ nitric oxide (NO); ↔ LH; ↔ testosterone (T)	↓ Pulsatility and resistive indices; ↑ testicular blood flow volume; ↑ testicular length and volume; ↓ echogenicity → active spermatogenesis	↑ Sperm concentration and viability	[30]

**Explanation for Arrows: ↑**: Increase in parameter. **↓**: Decrease in parameter. **↔**: No significant change.

## 5. Reproductive Responses in Female Non-Ruminants

In non-ruminant livestock species such as pigs, donkeys, and rabbits, inhibin immunization has shown varying degrees of success in improving reproductive outcomes. These findings, including the effects of inhibin immunization on male non-ruminant reproductive performance, are summarized in Table 3, which outlines the hormonal changes, testicular responses, and semen quality outcomes across these species

### 5.1. Sows

In swine, inhibin plays a significant regulatory role in ovarian folliculogenesis, influencing granulosa cell proliferation and endocrine function [90,91]. Polymorphisms within the inhibin gene complex have been linked to follicular cyst formation, highlighting its importance in maintaining normal ovarian physiology [92]. Immunization against inhibin has consistently increased ovulation rate and litter size in sows [93,94], and early-life immunization protocols have shown additional benefits. Pre-weaning immunization enhances live litter size but may slightly reduce the farrowing rate due to diminished luteal progesterone output. This effect may result from endocrine alterations caused by inhibin immunoneutralization, which elevates FSH and may disrupt the physiological balance between FSH and LH required for optimal corpus luteum development and progesterone secretion. Consequently, insufficient luteal progesterone during early gestation may compromise pregnancy establishment. Administering human chorionic gonadotropin (hCG) on day 5 post-insemination effectively restores luteal support and improves conception, providing a balanced approach for enhancing follicular development while maintaining adequate progesterone during early gestation [95]. Complementary in vitro findings demonstrate that exposure of porcine granulosa cells to anti-inhibin antibodies increases estradiol production, cell proliferation, and remodeling of extracellular-matrix and growth-factor pathways, reinforcing the concept that inhibin neutralization improves ovarian function at both systemic and local intra-ovarian levels [96].

Beyond follicular regulation, targeted luteal-support strategies are particularly important in immunized gilts. Evidence from hCG-treated gilts shows that corpus luteum lifespan, luteal angiogenesis, and endometrial vascular development can be enhanced even when circulating progesterone remains low, ultimately reducing early embryo loss and improving pregnancy maintenance [97]. Genetic studies further reveal that a 283 bp INHA insertion/deletion polymorphism is significantly associated with ovarian follicular cysts, likely due to impaired inhibin biosynthesis or signaling, contributing to altered FSH feedback and disrupted granulosa cell function [92]. The detailed effects of inhibin immunization on reproductive parameters in non-ruminants, including changes in antibody titers, FSH, and reproductive outcomes, are summarized in Table 3.

### 5.2. Mares and Jennies

In mares, passive immunization against inhibin has been shown to elevate circulating FSH and estradiol levels, stimulate follicular development, and increase ovulation rate, highlighting the regulatory role of inhibin in equine folliculogenesis [98]. Immunization against inhibin has also been reported to induce the development of multiple follicles and increase ovulation and embryo recovery rates in mares used for embryo transfer programs [99]. In pony mares, immunization against the recombinant porcine inhibin α-subunit increased anti-inhibin antibody titers and elevated plasma FSH and estradiol concentrations, resulting in a significant increase in the number of ovarian follicles during the estrous cycle, although ovulation rate remained unchanged [100]. In Dezhou jennies, active inhibin immunization induced a limited hormonal response, with 1.5 mg increasing plasma FSH, while 3 mg had no clear effect on FSH, progesterone, AMH, or estradiol [101]. Recent studies on inhibin immunization in mares and jennies (female donkeys), remain limited. Further research is needed to evaluate its effects on conception rate and overall reproductive performance.

**Table 3:** Effects of inhibin immunization on reproductive parameters in female non-ruminants.

Species	Immunogen Type	Antibody Titer/FSH Change	Reproductive Outcome	Key Findings	Reference
Pig (sows)	Pre-weaning immunization with recombinant inhibin-α (7 days before weaning); subset received 1000 IU hCG on day 5 post-insemination	Activin suppresses P4 synthesis; hCG stimulates P4 and reverses activin-induced inhibition of StAR, Cyp11a1, and 3β-HSDII in luteinized granulosa cells	Inhibin immunization ↑ live litter size but slightly ↓ farrowing rate; adding hCG tended to restore farrowing rate and sustain improved litter size	Inhibin immunization enhances follicular development, while post-insemination hCG rescues luteal progesterone production and supports early pregnancy.	[95]
Mare	Passive immunization (inhibin antiserum raised in castrated goat)	Significant increase in plasma FSH concentrations (dose-dependent)	Multiple ovulations (100 mL: 3.75 ± 0.63; 200 mL: 4.50 ± 0.65), increased follicular development	Inhibin neutralization enhanced FSH secretion, increased follicle growth, and resulted in multiple ovulations. Higher doses of inhibin antiserum resulted in more ovulations and increased estradiol-17β levels.	[98]
Sows	Inhibin (α-IF) fragment	Significant dose-dependent increase in antibody titer (*p* < 0.001);	Wean-to-service interval: reduced in higher dosages	Inhibin regulates FSH secretion post-weaning; blocking the acute FSH decrease had little effect on reproductive performance.	[102]
Pony mare (*Equus caballus*)	Recombinant porcine inhibin α-subunit (active immunization; three injections at 39-day intervals)	Significant increase in anti-inhibin antibody titer; plasma FSH and estradiol-17β concentrations significantly increased	Significant increase in small, medium, and large ovarian follicles; ovulation rate unchanged	Immunization stimulated follicular development during the estrous cycle, confirming the role of inhibin in regulating FSH secretion and follicular growth.	[100]
Dezhou jennies	Active immunization with inhibin (1.5 mg or 3 mg; primary + booster on days 1 and 23)	1.5 mg increased plasma FSH; 3 mg showed no significant effect on FSH, progesterone, AMH, or estradiol.	Limited endocrine response; no clear improvement in female reproductive hormones beyond FSH elevation at 1.5 mg	Active inhibin immunization in female donkeys produced a modest hormonal response, with 1.5 mg being more responsive than 3 mg.	[101]

**Note: ↑**: Increase; **↓**: Decrease.

### 5.3. Reproductive Responses in Male Non-Ruminants

In non-ruminant livestock species such as pigs, donkeys, and rabbits, inhibin immunization has shown varying degrees of success in improving reproductive outcomes. These findings, including the effects of inhibin immunization on male non-ruminant reproductive performance, are summarized in Table 4, which outlines the hormonal changes, testicular responses, and semen quality outcomes across these species.

#### 5.3.1. Boars

In boars, immunization strategies have predominantly focused on suppressing reproductive function rather than enhancing it, with early and repeated GnRH vaccination effectively inhibiting testicular steroidogenesis and reducing androstenone and skatole production, but also diminishing anabolic potential, nitrogen retention, and feed efficiency [103]. Active immunization against GnRH serves as a mechanistic opposite to fertility-enhancing inhibin immunization, as it induces functional castration by disrupting the central components of the hypothalamic–pituitary–gonadal axis. Han et al. [104] demonstrated that both GnRH vaccination and surgical castration decreased hypothalamic GnRH synthesis and content, downregulated Kiss1, GPR54, and androgen receptor expression, and suppressed circulating LH, FSH, testosterone, and inhibin B, leading to marked testicular atrophy and complete arrest of spermatogenesis. In contrast, surgically castrated boars exhibited elevated LH and FSH owing to loss of testosterone negative feedback, accompanied by reduced GnIH signaling. These findings highlight that while inhibin immunization seeks to selectively elevate FSH to stimulate gametogenesis, GnRH immunization exerts broad suppression across the entire HPG axis [104]. Given that most available data focus on fertility-suppressing GnRH vaccines rather than fertility-enhancing inhibin immunization, further research is needed to explore the potential benefits and reproductive applications of inhibin-targeted approaches in boars.

#### 5.3.2. Jacks

Recent studies have begun to explore the effects of inhibin immunization on male donkeys, particularly during the non-breeding season when endocrine activity is naturally suppressed. Akhtar et al. evaluated active immunization in 30 adult Dezhou jacks using recombinant porcine inhibin α-subunit (1.5 mg or 3 mg, primary + booster), which generated clear anti-inhibin antibody titers and modest increases in circulating FSH, LH, AMH, and activin A [40]. A subsequent study investigated endocrine responses in both sexes across the winter quiescent period. In jacks, the 3 mg dose stimulated noticeable increases in FSH, LH, testosterone, and AMH, indicating partial activation of testicular function despite the seasonal decline, whereas jennies receiving 1.5 mg showed only modest increases in FSH with no significant changes in progesterone, estradiol, or AMH [101]. Inhibin immunization research in donkeys is still in its early stages; further studies are needed to evaluate different doses, optimize vaccination intervals, and assess responses across various donkey breeds to establish reliable and effective protocols.

#### 5.3.3. Male Rabbits

Studies in rabbits provide additional evidence that inhibin immunization can mitigate environmentally induced reproductive decline. During periods of summer heat stress, control rabbits typically exhibit marked reductions in sperm concentration and motility; however, immunization with recombinant porcine inhibin-α (0.05 or 0.125 mg/kg) generated strong antibody titers after the second injection and produced sustained improvements in semen quality compared with non-immunized controls [105]. Complementary findings come from testosterone-targeted immunization studies, where active immunization during early puberty elicited robust anti-testosterone antibody responses that attenuated negative feedback on the hypothalamic–pituitary–testicular axis, leading to elevated LH and total testosterone, enlarged testes with Leydig cell hyperplasia, and upregulated expression of key steroidogenic enzymes and regulatory genes (P450scc, P450c17, 3β-HSD, GnRH, GnRH-R, LHβ, AR, ERα, Kiss-1, GPR54, inhibin-α/βA) without disturbing the morphology of the seminiferous epithelium or spermatogenesis [106]. Given that research on inhibin immunization in rabbits remains limited, further controlled studies are needed to validate these findings, optimize dosing strategies, and clarify long-term reproductive outcomes.

**Table 4:** Effects of inhibin immunization on male reproductive parameters in non-ruminant livestock.

Species	Immunogen Type	Hormonal Change	Testicular Response	Semen Quality	Reference
Boar	GnRH tandem-dimer peptide vaccine (immunocastration) vs. surgical castration	GnRH vaccine: ↓ LH, FSH, testosterone, inhibin B; surgical castration: ↑ LH, FSH, testosterone & inhibin B undetectable	Severe testicular atrophy; arrested spermatogenesis in both groups; reduced pituitary weight after GnRH vaccine, increased after surgical castration	Not evaluated; both interventions induce functional castration and infertility	[104]
Donkey (adult Dezhou jacks, non-breeding season)	Recombinant porcine inhibin α-subunit protein (3 mg or 1.5 mg; primary + booster; mineral oil adjuvant)	Slight ↑ FSH, ↑ LH, ↑ AMH, ↑ activin A; testosterone and progesterone largely unchanged	Transient ↑ spermatogonia and elongated spermatids (day 28); Sertoli cell vacuolation, empty lumina, apoptosis; seminiferous tubule diameter mostly unchanged	Not evaluated (study focused on plasma hormones and testicular histology)	[40]
Donkey (Dezhou jacks & jennies, non-breeding season)	Recombinant porcine inhibin α-subunit protein; 3 mg or 1.5 mg; primary + booster (days 1 and 23)	Males: 3 mg ↑ FSH, ↑ LH, ↑ testosterone, ↑ AMH. Females: 1.5 mg ↑ FSH; P4, AMH, E2 unaffected.	Males: endocrine activation only; no structural data reported. Females: ovarian hormones largely unchanged.	Semen quality not evaluated; study focused on plasma hormones only.	[101]
Rex rabbit	Recombinant porcine inhibin-α (0.05 mg/kg and 0.125 mg/kg; s.c.; 3 injections)	Significant rise in anti-inhibin antibody titers after second immunization	Likely relief of FSH suppression, improving spermatogenic activity	↑ Sperm concentration and motility during heat stress (*p* < 0.05); partial recovery of semen quality	[105]
Rabbit	Testosterone–3(O-carboxymethyl) oxime–BSA conjugate, 1 mg per rabbit s.c.; primary + booster at 4-week interval	Strong and sustained antitestosterone antibody response; ↑ LH and ↑ total testosterone; transient ↑ FSH after booster	↑ Testis weight and volume; Leydig cell hyperplasia and hypertrophy; ↑ testicular P450scc, P450c17, 3β-HSD protein and mRNA; ↑ testicular AR, inhibin-α and inhibin-βA; normal seminiferous tubules and spermatogenesis	Direct semen traits not measured; data indicate increased steroidogenic and spermatogenic capacity and likely improved sperm production later in life	[106]

**Explanation for Arrows: ↑**: Increase. **↓**: Decrease.

## 6. In Vitro Embryo Production and Integration with ARTs

Multiple studies demonstrate that inhibin immunization significantly enhances both the quantity and quality of embryos produced in vitro. In superovulated cattle, immunization increased total and transferable embryo numbers by approximately 90% and 65% [56], as well as 69% and 19% in another study [107]. Supplementation of polyclonal anti-inhibin antibodies into buffalo and bovine in vitro maturation (IVM) media improved oocyte maturation and cleavage rates following IVF [24,31]. Increasing antibody concentration from 50 µg/mL to 100 µg/mL elevated bovine oocyte maturation from 72.7% to 80.3%, and raised cleavage rates from 87.5% to 96.7% [24]. Molecular evidence reveals that inhibin expression patterns in oocytes correlate with follicular development stage; in pigs, inhibin βA and βB expression was significantly higher in oocytes from larger follicles, suggesting an association with oocyte maturation and predictive value for IVF outcomes [108]. In dromedary camels, inhibin immunization improved ovarian follicular activity and mitigation of seasonal anestrus, ultimately increasing the efficiency of IVF and ICSI [109]. Evidence from mice demonstrates that neutralizing free inhibin α-subunit decreased ovulation by 47.29% but enhanced fertilization by 55.68% and blastocyst formation by 43.85%, suggesting that free α-subunit may antagonize mature inhibin’s regulatory role and influence oocyte and embryo development [110]. When integrated with MOET, Ovsynch, and IVF/ICSI protocols, inhibin immunization has low-quality evidence, reflecting the limited research conducted in this species. A structured evaluation of these findings using the GRADE framework is presented in Table 5, highlighting the need for more rigorously designed and species-specific studies to strengthen translational application.

## 7. Challenges and Future Perspectives

Although inhibin immunization has demonstrated promising potential for improving reproductive performance in livestock, several scientific and practical challenges must be addressed before its large-scale commercial application. A primary priority is the development of standardized immunization protocols that can be reliably applied across different production systems and animal species. Current studies vary considerably in vaccine formulation, dosage, immunization schedule, and physiological stage of the animals, which complicates comparisons and limits the establishment of universal guidelines. Long-term investigations evaluating reproductive performance across multiple estrous cycles are also necessary to determine the sustainability and stability of immunization effects.

Further research should focus on clarifying the mechanisms underlying the FSH–inhibin–gonadal axis and the species-specific responses to inhibin immunization. Molecular investigations examining gonadal gene expression, follicular development, oocyte competence, and semen quality biomarkers may provide deeper insight into the physiological basis of fertility improvements. Additionally, studies evaluating embryo development, offspring viability, and long-term developmental outcomes following embryo transfer from immunized donors are necessary. Economic feasibility, regulatory considerations, and integration with assisted reproductive technologies should also be explored to support practical application in livestock production.

### 7.1. Species-Specific Challenges

Species-specific variations in reproductive physiology significantly influence responses to inhibin immunization and must therefore be considered during protocol development. Ruminant species such as cattle and sheep generally exhibit consistent and well-characterized responses, with clear relationships between antibody titers, FSH elevation, and follicular development. These species currently represent the most reliable candidates for practical application of inhibin immunization.

Goats also demonstrate favorable responses, particularly when immunization is performed under subluteal progesterone conditions that support follicular recruitment. In contrast, pigs present distinct physiological challenges. Inhibin neutralization can elevate circulating activin A levels, which may suppress corpus luteum progesterone production and compromise pregnancy maintenance. Consequently, luteal support strategies are often required in porcine protocols.

Camelids show unique ovarian responses characterized by sustained follicular stimulation and atypical patterns of ovarian activity, which require further mechanistic investigation before practical application can be recommended. Evidence in non-ruminant species such as donkeys and rabbits remains limited, and current data are insufficient to establish clear immunization strategies or commercial recommendations for these animals.

### 7.2. Key Safety Concerns

While inhibin immunization demonstrates considerable reproductive benefits, emerging evidence indicates several important adverse effects warranting careful consideration. Most critically, elevated activin A concentrations consequent to inhibin neutralization suppress corpus luteum progesterone synthesis, a phenomenon particularly problematic in porcine species. Gilts receiving high-dose anti-inhibin antibodies exhibited reduced farrowing rates ranging from 75–80% compared with 85–90% in controls, with embryonic and fetal loss rates of 18–25% in the absence of concurrent luteal support [95]. These findings necessitate mandatory human chorionic gonadotropin administration or alternative luteal support strategies in porcine inhibin immunization protocols.

In addition, the dose-dependent effects of inhibin immunization have not yet been fully characterized. Excessively high antibody titers may interfere with final oocyte maturation and compromise gamete quality. Furthermore, long-term reproductive consequences across multiple breeding cycles remain largely unknown. In avian species, some studies have reported testicular dysfunction in male geese following inhibin immunization, suggesting that avian reproductive endocrine regulation may differ substantially from mammalian FSH–inhibin feedback systems.

Beyond physiological safety, animal welfare considerations must also be taken into account. Repeated immunization procedures and associated hormonal manipulations may influence stress responses and overall physiological balance, particularly in intensive production systems. Therefore, future research should carefully evaluate welfare implications while optimizing immunization strategies to ensure safe, ethical, and sustainable implementation of inhibin immunization in livestock reproduction.

## 8. Conclusions

Inhibin immunization effectively enhances reproductive performance in livestock by relieving FSH suppression, thereby increasing follicular recruitment, ovulation rates, and embryo yield in females, and improving spermatogenic capacity and semen quality in males. Integration with MOET, synchronization protocols, and IVF/ICSI substantially amplifies breeding program efficiency. However, species-specific variability, dose-dependent effects on luteal function, and limited long-term fertility data necessitate further optimization. Future investigations must establish standardized protocols, evaluate repeated immunization across reproductive cycles, characterize gonadal gene expression profiles, and assess offspring viability to establish inhibin immunization as a reliable, economically viable reproductive biotechnology for commercial livestock systems.

## Figures and Tables

**Table 5:** Evidence quality assessment using the GRADE framework for inhibin immunization outcomes across species.

Outcome	Species	Evidence Quality
Ovulation rate	Cattle, Sheep, Goats	Moderate
Embryo yield	Cattle	Moderate
Sperm quality	Bulls, Bucks	Low—moderate
Spermatogenesis	Donkeys, Rabbits	Low
Fertility outcomes	Camels	Very low

## Data Availability

No new data were created or analyzed in this study. Data sharing is not applicable.
